# Localization and characterization of thyroid microcalcifications: A histopathological study

**DOI:** 10.1371/journal.pone.0224138

**Published:** 2019-10-24

**Authors:** Joanne Guerlain, Sophie Perie, Marine Lefevre, Joëlle Perez, Sophie Vandermeersch, Chantal Jouanneau, Léa Huguet, Vincent Frochot, Emmanuel Letavernier, Raphael Weil, Stéphan Rouziere, Dominique Bazin, Michel Daudon, Jean-Philippe Haymann

**Affiliations:** 1 Sorbonne Université, INSERM, UMR_S 1155, AP-HP, Hôpital Tenon, Paris, France; 2 Service d’Oto-rhino-laryngologie et de Chirurgie Cervico-Faciale, Hôpital Tenon, Paris, France; 3 Service d’Anatomopathologie, Hôpital Tenon, Paris, France; 4 Service d’Explorations Fonctionnelles Multidisciplinaires, Assistance Publique—Hôpitaux de Paris (AP-HP), Hôpital Tenon, Paris, France; 5 Laboratoire de Physique des Solides, UMR CNRS 8502, Université Paris Sud, Université Paris Saclay, Orsay Cedex, France; 6 Laboratoire de Chimie Physique, Université Paris Sud, Orsay, France; Central University of Rajasthan, INDIA

## Abstract

Thyroid calcification is frequent in thyroid nodules. The aim of our study was to evaluate the prevalence of calcifications in thyroid tissue samples of patients with various thyroid diseases, and to identify their composition according to their localization. Among 50 thyroid samples included, 56% were malignant (papillary carcinoma) and 44% were benign (adenoma, multinodular goiter, Graves’ disease, sarcoidosis). Calcifications were found in 95% of samples using polarised light microscopy, whereas only 12% were described in initial pathological reports. Three types were individualised and analyzed by infrared spectrometry (μFTIR): colloid calcifications composed of calcium oxalate, capsular calcifications and psammoma bodies, both composed of calcium phosphate. Of notice, psammoma bodies characterized by FE-SEM were composed of concentric structure suggesting a slow process for crystal deposition. Calcium phosphates were found only in malignant samples whereas calcium oxalate was not associated with a define pathology. Proliferation assessed by KI67 staining was high (33% of positive follicles), and RUNX2, OPN, and CD44 positive staining were detected in thyrocytes with a broad variation between samples. However, thyrocyte proliferation and differentiation markers were not associated with the number of crystals. TRPV5 and CaSR expression was also detected in thyrocytes. mRNA transcripts expression was confirmed in a subgroup of 10 patients, altogether with other calcium transporters such as PMCA1 or Cav1.3. Interestingly, TRPV5 mRNA expression was significantly associated with number of colloid calcifications (rho = -0.72; p = 0.02). The high prevalence of calcium oxalate crystals within colloid gel raises intriguing issues upon follicle physiology for calcium and oxalate transport.

## Introduction

Calcifications are frequently detected in thyroid tissue by pathologists. However, crystal composition and/or pathophysiological processes have been poorly investigated as no clinical relevance was reported either for diagnosis or prognosis.

Thyroid nodules are very common in the population and about 5% of them are malignant [1] with a prevalence of calcification in around 40% of malignant nodules and 20% of benign nodules [2]. Thyroid ultrasound of micro and macrocalcifications are indeed well described in the literature. TIRADS scoring (Thyroid Image Reporting And Data System) is frequently used in clinical practice as risk factor for thyroid lesions [3]: microcalcifications are predictive of malignancy [2,4] whereas central macrocalcifications are usually predictive of benign pathology. However, several diseases may be associated with calcifications such as thyroid papillary carcinoma, nodular goiters or Graves’ disease, and despite several studies, no clear association between calcifications and pathology was demonstrated [5–10], (conversely to microcalcifications in cervical lymph nodes which are predictive of thyroid papillary carcinoma metastasis [11]). One caveat is due to the fact that papillary carcinoma (and particularly microcarcinoma) is frequently incidental, associated with other pathologies such as Grave’s disease or nodular goiters [12].

At the microscopic scale, three types of thyroid calcifications are described [13]: 1) Psammoma bodies presenting as round and lamellar calcification which do not polarize; 2) Capsular calcification usually described as unspecific eggshell calcifications of various sizes, surrounding the capsule; 3) Colloid calcifications within colloid of follicles, presenting as birefringent crystals under polarized light microscopy [5]. Crystals within follicle colloid were reported only in human thyroids [6,14] and identified as calcium oxalate [6], whereas hydroxyapatite was found in almost all macroscopic calcifications from thyroid tissue extracts [7].

Our focus, here, was to study the prevalence of calcifications in thyroid tissue sample of patients with various thyroid diseases, and to identify their composition according to their localization.

## Materials and methods

### Samples

#### Materials and participants

All formalin-fixed paraffin-embedded (FFPE) samples from partial or total surgical thyroidectomy collected during six months (between July and December 2014) were included in the study. Ten frozen thyroid samples collected during the year 2016 were also included for mRNA study.

**Fig 1:**
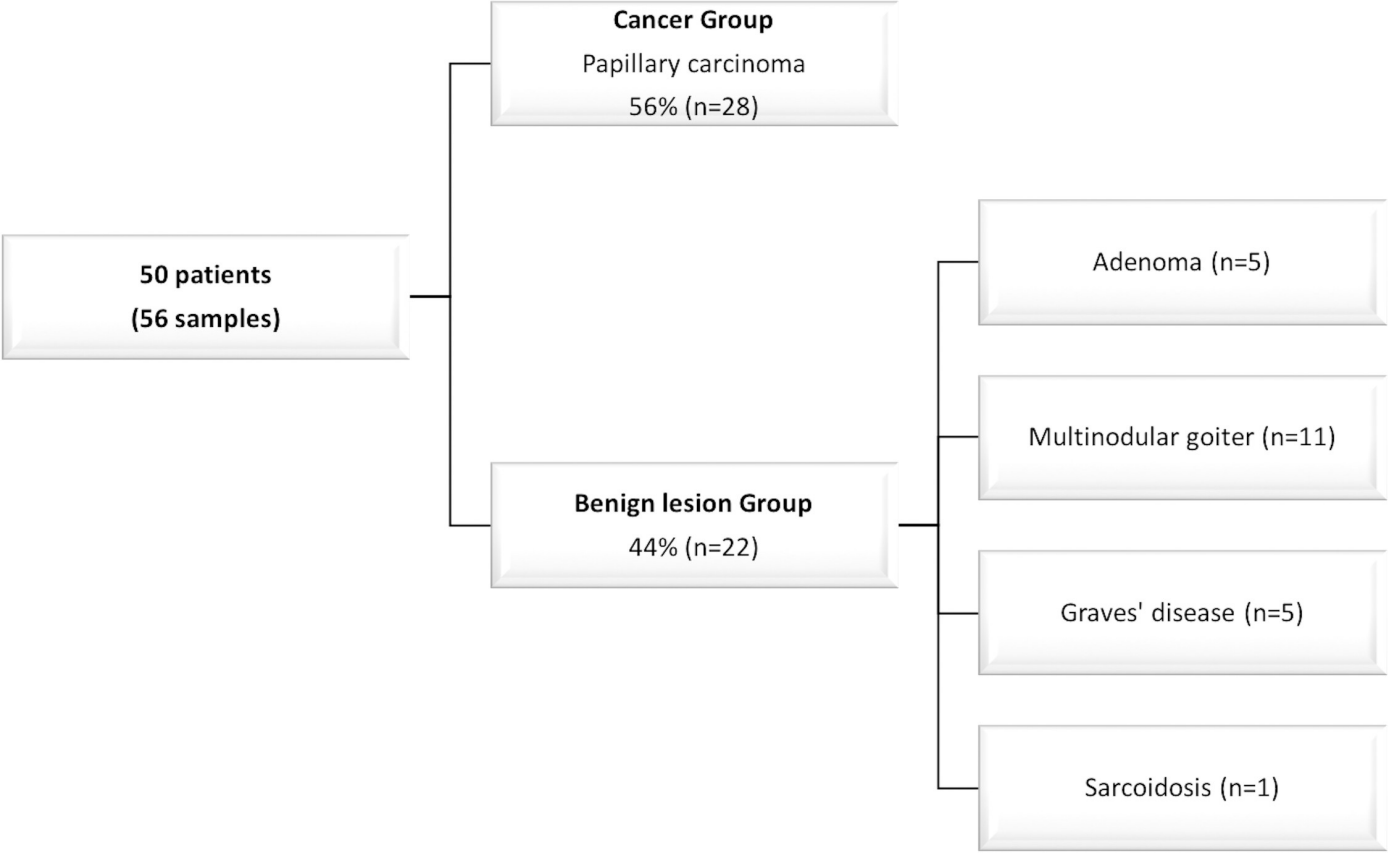
Patients characteristics according to thyroid disease.

Fifty patients were included and six patients were operated twice during the 6 months for completion thyroidectomy. Partial thyroidectomy (n = 28) and total thyroidectomy (n = 28) were included (Fig 1).

#### Ethical consideration

The study was approved by the Commission d’Evaluation et de Recherche Observationnelle en OtoRhinoLaryngologie (CEROL) (Ethics Committee of the Society of Otolaryngology, France). In accordance with French legislation (Public Health Code amended by law No. 2004–806, August 9, 2004 and the Huriet-Sérusclat Law 88–1138, December 20, 1988), a patient information letter was issued and verbal informed consent was obtained. Data were strictly anonymous.

#### Sample preparation

Slices were prepared from FFPE blocks. For each patient, focal lesion such as adenoma, papillary carcinoma (with follicular or papillary architecture) or microcarcinomas were analyzed. Blocks from diffuse lesion encountered in multinodular goiter, Graves’ disease, or sarcoidosis were randomly selected and analyzed. FFPE blocks were sectioned with a microtome (Microtom HM 325, ThermoFisher Scientific, Waltham, Massachusetts, United States) to obtain 4μm thick sections. Three consecutive sections were realized for each block and stained for Von Kossa stain (Von Kossa, to identify and localize calcium deposits in paraffin sections), and Hematoxylin and Eosin stain (HE) and one section was analyzed using FE-SEM (Zeiss Supra 55VP Field Emission Scanning Electron Microscope) and micro Fourier transform infrared spectrometry (μFTIR) (Spotlight 400 infrared imager, Perkin-Elmer, Villebon-sur-Yvette, France). Others sections were stained on SuperFrost® for immunohistochemistry.

### Identification and characterization of calcium deposits

#### Morphological study

Calcium deposits were examined by normal and polarized light microscopy to determine their size, shape, occurrence, distribution and location, on HE and on Von Kossa slices. Calcifications were counted on the HE slice with polarized light, on five random fields (x100 magnification).

Composition of calcifications was performed by μFTIR. Data were collected in the reflection mode between 4000 and 700cm^-1^, with a resolution of 4 cm^-1^ [15]. Microcalcifications were further characterized by FE-SEM (Zeiss Supra 55VP Field Emission Scanning Electron Microscope) in order to describe their morphology at the micrometer scale with a 3D reconstruction [16]. Energy dispersive x-ray analysis (EDX) was used for some samples for the elemental analysis [17].

#### Immunohistochemistry and RT-PCR

After paraffin removal, antigen-retrieval, and incubation with a peroxydase blocking solution and bovine serum albumin (BSA) to reduce background staining, sections were incubated with one of the following primary antibodies overnight at 4°C: anti-TTF1 (Dako,Glostrup, Denmark), KI67, osteopontin, RUNX2, TRPV5, calcitonin (Abcam, Paris, France), anti-CD44 (Exbio, Prague, Czech Republic) and anti-CaSR (Fisher, Illkirch, France). Sections were revealed by a Biotin-Free Polyvalent AEC secondary antibody (3-amino-9-ethylcarbazole, Dako; Glostrup, Denmark). Counterstaining was performed with HE. Negative controls were performed by omitting the primary antibody.

Proliferation index was performed for each slice on ten random fields with x400 magnification and defined as the ratio of the number of vesicles with at least more than 10% of KI67 positive thyrocytes/total number of vesicles.

Snap frozen samples from ten patients collected in the operating room were used for RT-PCR and HE staining. A normal frozen human kidney sample was used as control. Total RNA purification was performed using Trizol-chloroform protocol according to the manufacter’s instructions. cDNA were synthesized from 1μg of total RNA in 20μL with a reverse transcriptase (ThermoFisher Scientific, Waltham, Massachusetts, United States). Amplification was done with SYBR Green (SensiFast SYBR, Bioline, London, United Kingdom) and sequence specific primers by thermocycler (Light Cycler 480, Roche, Bale, Switzerland). The following protocol was used: 95°C for 5 minutes then 45 cycles at 95°C for 15 seconds, 60°C for 15 seconds and 72°C for 15 seconds. All samples were normalized to housekeeping gene expression human 18S rRNA. The primers sequences are detailed in Table 1.

**Table 1:** Primer sequences for mRNA transcripts.

Human TRPM6	Sense strand	hTRPM6-3var-s	AAGGACTCCAGGTGCCAAT
	Antisense strand	hTRPM6-3var-as	GCTGTACTCCTCTTCAGAGATGC
Human VDR	Sense strand	hVDR-3var-s	GACCTGTGGCAACCAAGACT
	Antisense strand	hVDR-3var-as	GAACTTGATGAGGGGCTCAA
Human NCX1	Sense strand	hSLC8A1-4var-s	GCCCTTGTGGTTGGGACT
	Antisense strand	hSLC8A1-4var-as	CCACATTCATCGTCGTCATC
Human PMCA1	Sense strand	hATP2B1-2var-s	TCCAGAAGGGGATAATGCAC
	Antisense strand	hATP2B1-2var-as	TCAATCCAACCAGTTTCACCT
Human Calgranulin B	Sense strand	hS100A9s	GTGCGAAAAGATCTGCAAAA
	Antisense strand	hS100A9as	TCAGCTGCTTGTCTGCATTT
Human TRPV5	Sense strand	hTRPV5s	AGATCGACTCCTGGGGAGA
	Antisense strand	hTRPV5as	TGGGGTCTGTTCCAGAATTT
Human TRPV6	Sense strand	hTRPV6s	AGAGCCGAGATGAGCAGAAC
	Antisense strand	hTRPV6as	GAAGGAGAGGAGACTCCCAGA
Human MGLA	Sense strand	hMGP2vars	TCACATGAAAGCATGGAATCTTA
	Antisense strand	hMGP2varas	ACAGGCTTAGAGCGTTCTCG
Human Cav1.3	Sense strand	hcav1.3_3vars	TTTGGTTCGAACGGCTCTTA
	Antisense strand	hcav1.3_3varas	AGCCCGAAGTTCTTCATTAGC
Human CaV1.3 var 1	Sense strand	hCASRvar1s	CGAGGAGAAAATCCTGTGGA
	Antisense strand	hCASRvar1as	AGTTGGAGAAGGGCACCTG
Human CaV1.3 var 2	Sense strand	hCASRvar2s	CTTTCGCGCAGGAGAGTG
	Antisense strand	hCASRvar2as	GCCTCCTGTGATGCCTTTC
Human Ostéopontin	Sense strand	hSPP1s	AGGACTCCATTGACTCGAACGA
	Antisense strand	hSPP1as	CACCTCGGCCATCATATGTGT
Human Calcitonin	Sense strand	hCALCAs	CACGCTCAGTGAGGACGAAG
	Antisense strand	hCALCAas	AAGTCCTGCGTGTATGTGCC

#### Statistical analysis

Results are expressed by median and interquartile range. Comparison between different groups were performed using a nonparametric Wilcoxon-Mann-Whitney test for quantitative variables and a Chi2 test for qualitative variables (BiostaTGV software). A non parametric Spearman test was used for correlations. Significance was defined as p<0.05.

#### Results

Among our population, 38 patients were female and 12 were males with a median age of 49 [40–54] years. As shown in Fig 1, papillary carcinoma was diagnosed in 28 thyroids. 28% (n = 8/28) were microcarcinoma and 58% (n = 16/28) were multiple foci (from two to twelve foci). This population was thus referred as a “Tumor group” and compared to a “non Tumor group” of 22 patients including 5 adenomas, 11 multinodular goiters, 5 Graves’ diseases and one sarcoidosis. Of notice, in the Tumor group, papillary, papillo-follicular and papillo-oncocytic architecture were encountered in 16, 9 and 3 cases respectively. Moreover, aside the detection of carcinoma, a multinodular goiter was present in 6 patients, one patient had Graves’ disease and three had an associated vesicular adenoma.

### Morphologic study

#### Thyroid calcifications identification and localization

Among 56 analyzed samples, the presence of crystal deposits detected by the pathologist was reported only in 7 cases: psammoma were detected in 3 cases and colloid calcified nodules in 4 cases. After careful proofreading by our pathologist on FFPE slices without polarized microscopy, psammoma bodies were confirmed in 3 samples (5%), calcifications outlying the nodules were found in 5 samples (9%). Of notice, colloid calcifications were detected in 4 samples, however under polarized light microscopy, they were found in 51 out of 56 samples (i.e. 91%). Overall 95% of the samples had at least one of the three types of calcification described above.

**Fig 2:**
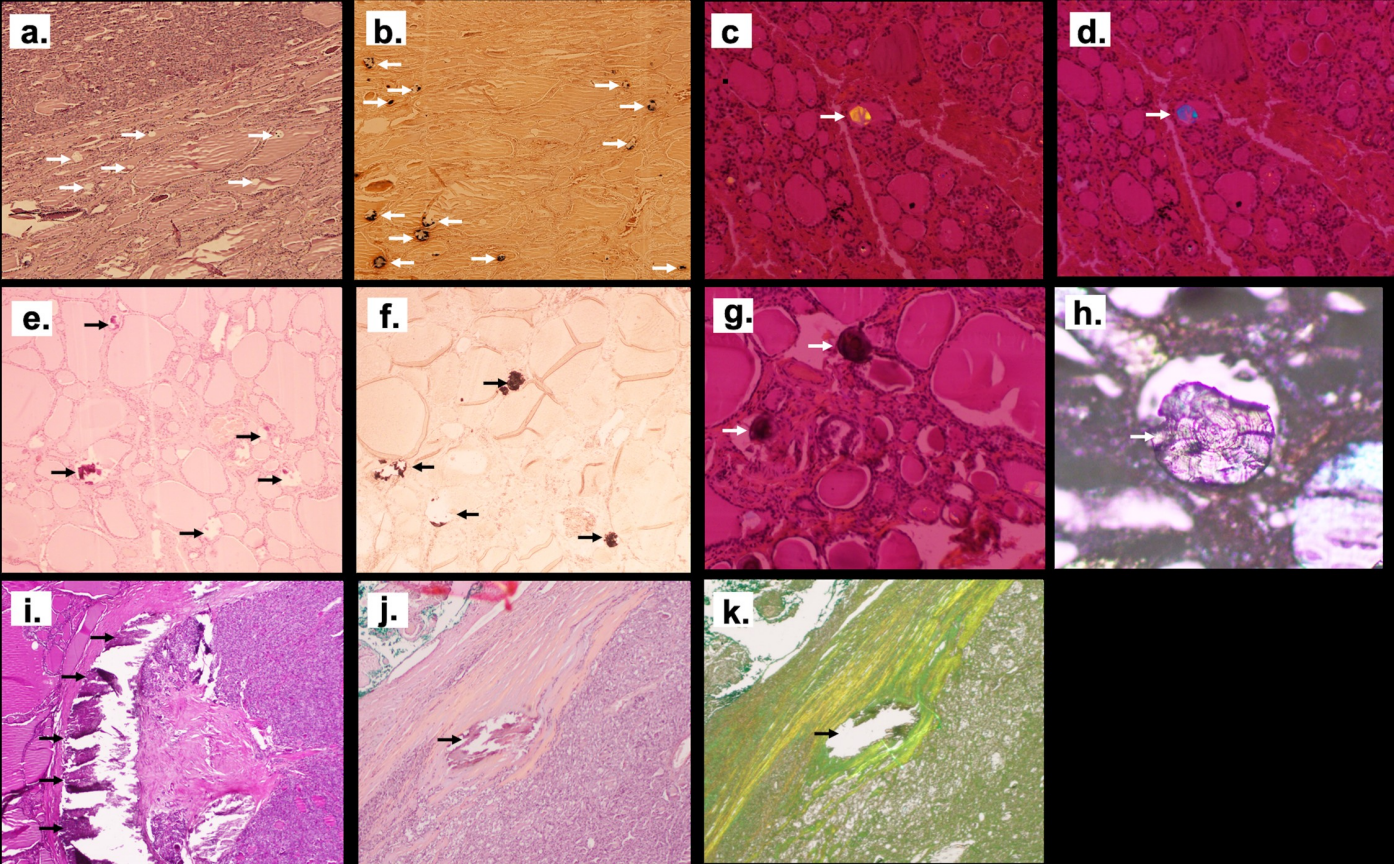
Illustration of the different patterns of crystals deposits in thyroid tissue using optic microscopy. Crystals located were misdiagnosed by Hematoxylin and Eosin staining (HE) (arrows show the location of colloid crystals that are misdiagnosed by HE staining in (a), but detected by Von Kossa staining as dark composits (arrows show the black staining of crystals with VK coloration inside the vesicles in (b) and also by polarized light microscopy (arrows show the birefringent character of colloid crystals in (c) and (d), magnification x40). Psammoma bodies were detected both by HE (arrows show the HE staining of psammoma bodies outside the vesicles in (e), and Von Kossa staining (arrows show the black staining of psammoma bodies with VK coloration in (f) with no polarized property and (g) psammoma bodies shown with arrows do not have birefringent character). A lamellar aspect was detected on silver coated slide without staining (the arrow show a psammoma body in (h)) (x200 magnification). Large calcification in nodule’s capsule were easily detected with HE (in (i) and (j) capsule calcification are shown with arrows) and also on silver coated slide without staining (an arrow shows in (k) the same capsule calcification than (j) in a silver coated slide without staining) (x40 magnification).

As shown in Fig 2A, 2B, 2C and 2D, colloid calcifications may be detected by Von Kossa staining or by polarized light microscopy and not by hematoxylin eosin staining. These calcifications are located in the follicles and are birefringent under polarized light. At least one colloid calcification per slice was detected in 91% of cases. However, calcifications number may vary considerably between the different samples with a mean number of 50 (+/-97) and a median number of 11 [2–44]. Colloid crystal size was also variable with a median diameter of 16 μm (from 1 to 374μm).

As shown in Fig 2E, 2F, 2G and 2H, psammoma bodies were detected by Von Kossa or HE staining in the interstitium outside follicles’ colloid, as round, lamellar and concentric calcifications with no polarization property. Median diameter was 100 μm [75–116].

Last, calcifications outlying nodules in the capsules were very large, easily detected by hematoxylin and eosin staining, with no polarization (Fig 2I, 2J and 2K).

#### Composition of thyroid crystals

A subgroup of 27 patients was studied using a μFTIR spectrometry imager: 18 patients in the tumor group and 9 patients in the non tumor group. Overall, 132 calcifications were analyzed and classified according to their tissue localization: colloid, interstitial or capsular calcifications (Table 2). All colloid calcifications (100%, n = 104) were composed of calcium oxalate with calcium oxalate monohydrate (COM) in 58% of cases, calcium oxalate dihydrate (COD) in 14% and a mixture in 28% of cases (COM + COD). No calcium phosphate was detected even in small amount. Conversely, all psammoma bodies (100%, n = 24) were composed of calcium phosphate, the most frequent component was carbonated calcium apatite (CA) (88% of cases) either alone (30%) or mixed with a mixture of amorphous carbonated calcium phosphate (ACCP) and CA (58%). As shown Table 2, other calcium phosphates such as whitlockite could also be detected. Of notice, capsule calcifications were also calcium phosphates in all samples (100%, n = 5), with also the presence of CA, ACCP and whitlockite compounds.

**Table 2:** Composition of crystal deposits in thyroid samples according to their location.

	n (= 132)	Calcium oxalate			Calcium Phosphate			
		COM	COD	COM+ COD	CA	ACCP	CA + ACCP	Other
Colloid calcifications	104	58%	14%	28%	0	0	0	0
Psammoma bodies	24	0	0	0	30%	8%	58%	4%*
Capsule calcifications	4	0	0	0	25%	0	50%	25%*

COM = calcium oxalate monohydrate. COD = calcium oxalate dihydrate. CA = carbonated calcium apatite. ACCP = amorphous carbonated calcium phosphate.

*One psammoma body was composed of CA with Whitlockite.

**One capsule calcification was a mixture of CA, ACCP and Whitlockite.

#### Morphology of thyroid crystals

**Fig 3:**
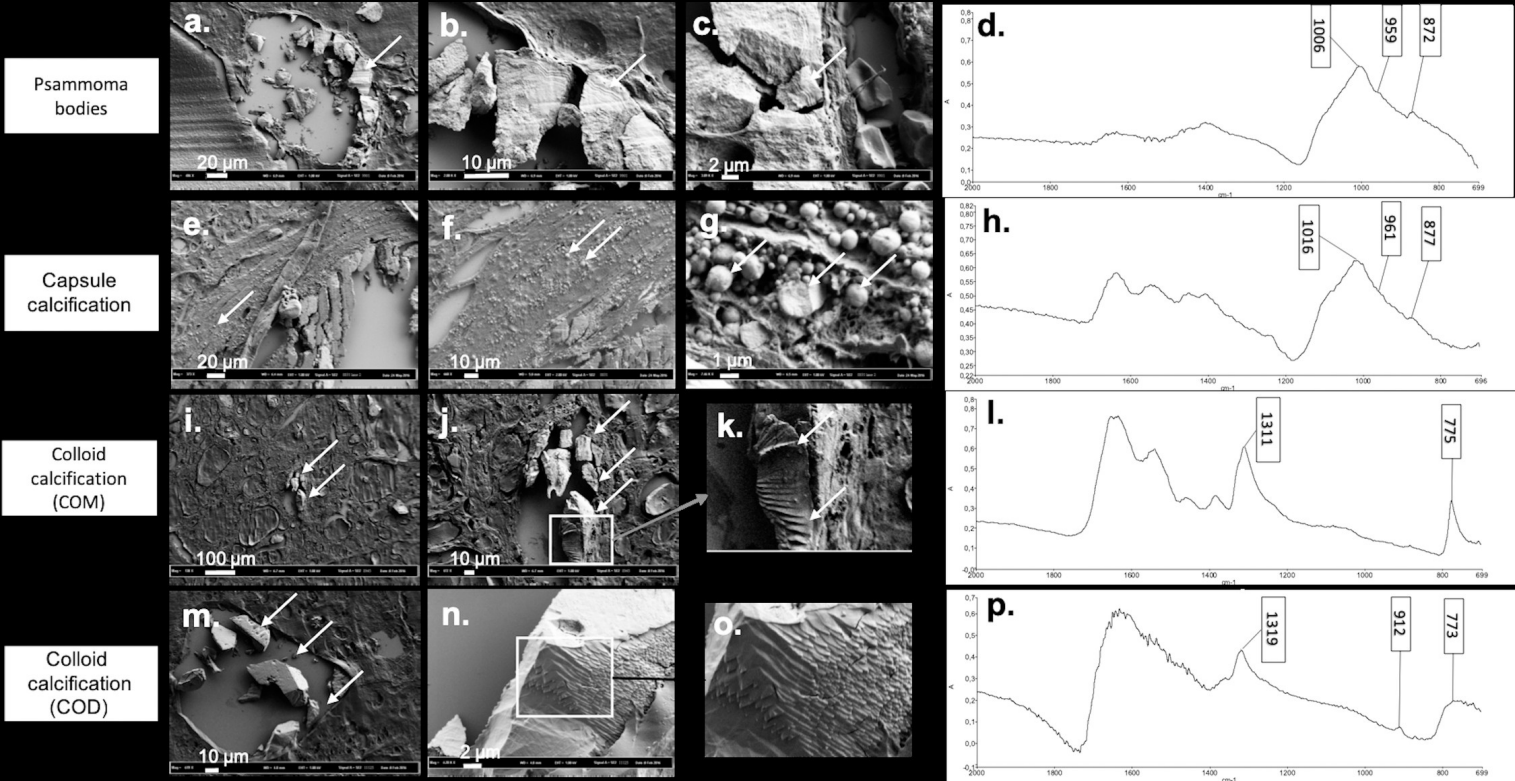
Illustration of the different patterns of crystals deposits in thyroid tissue using FE-SEM according to composition assessed by μFTIR spectrometry. (a) (b) (c) Psammoma bodies with presence of growth lines in FE-SEM (a) arrow shows the psammoma body in FE-SEM, (b) and (c) arrows indicate the growth lines on the same psammoma body shown in (a), with a higher magnification), (d) FTIR absorption spectrum showing a mixture of CA and ACCP. (e) (f) (g) Capsule calcifications detected as an agglomeration of small spherules by FE-SEM (arrows show the same capsule calcification with different magnifications). (h) FTIR absorption spectrum showing a mixture of CA and ACCP. (i) (j) (k) COM, assessed by FTIR absorption (arrows show the same COM crystal on the three images with different magnifications). (l) appearing as overlapped sticks by FE-SEM. (m) (n) (o) COD with a pattern looking close to COM structure with overlapped sticks and sometimes perpendicular intersects (arrows show the COD crystals with different magnifications). (p) FTIR absorption spectrum of COD crystal.

In order to detect at the micrometer scale crystal morphology according to chemical composition, we processed and analyzed both on FE-SEM and μFTIR spectrometry forty-five different samples containing crystallites. Fig 3 illustrates representative results. Psammoma bodies appeared as concentric and lamellar structures (Fig 3A, 3B and 3C) with peripheral striations suggesting growth lines. Calcium phosphate compounds did not exhibit the usual spherical morphology described in kidney or prostate gland [18,19].

Conversely, aggregated small spheres of different size (at micrometer scale) were detected in capsular calcifications (Fig 3E, 3F and 3G) with an internal radial structure (Fig 3G).

As shown in Fig 3, colloid calcifications composed of pure COM (Fig 3I, 3J and 3K) or pure COD (Fig 3M, 3N and 3O) had an overlap sticks appearance whereas COD is usually described as bipyramidal structure in urine. The presence of calcium was further confirmed using electron diffraction X-ray microscope (EDX, S1 Fig), thus ruling out other cations associated with oxalate crystallites.

### Pathophysiological processes

#### Colloid calcification and thyrocytes’ phenotypes

**Fig 4:**
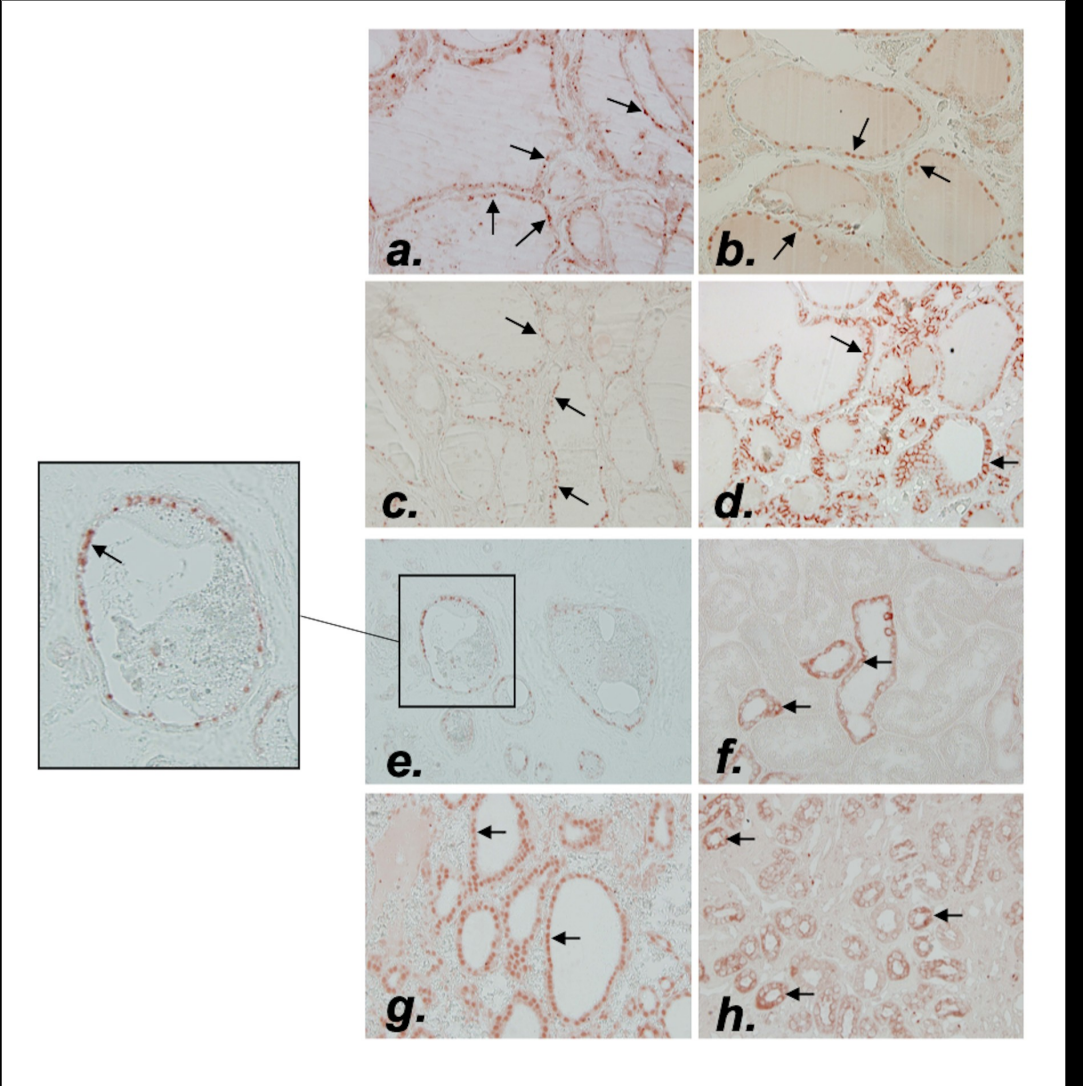
Immunochemistry of Ki67, RUNX2, OPN, CD44, TRPV5 and CaSR in thyroid sample. Representative illustration of Ki67 (a) (arrows indicating Ki67 positive cells), RUNX2 (b) (arrows indicating RUNX2 positive cells), OPN (c) (arrows indicating OPN positive cells) and CD44 staining (d) (arrows indicating CD44 staining) in thyroid samples. TRPV5 and CaSR staining in thyroid sample (e) and (g) respectively (arrows indicating the TRPV5 and CaSR positive cells respectively) compared to a normal kidney sample (f) and (h) respectively. Magnification x100 and x400.

We next focused on colloid calcifications in order to better understand the pathophysiological process leading to the onset of COM and COD crystals in most biopsies. Indeed, thyroid vesicles are a confined space containing colloid material, surrounded by thyrocytes and a basement membrane. In all tested samples, TTF1 was expressed on thyrocytes; however, proliferation was unexpectedly high, with a positive KI67 staining in 33% of vesicles [6%-54%], (minimum and maximum respectively: 0% and 89%) (Fig 4). Moreover, as shown in Fig 4, positive RUNX2, OPN, and CD44 staining were also detected in thyrocytes with a broad variation between samples and no obvious significant staining intensity between the tumor and non tumor group (S3 Table). Of notice, no link was found for all the biomarkers mentioned above and colloid calcification number.

#### Thyrocyte expression of calcium transporters

Calcium transporters expression was studied by RT-PCR in a subgroup of ten frozen thyroid samples. As shown in Table 3, several calcium transporters expressed in the kidney tissue were also expressed at variable but significant levels in the thyroid samples such as TRPV5, PMCA1 or Cav1.3, but also the calcium sensor CaSR, and the calcitriol receptor VDR (Table 3). The small number of samples with only 3 samples in the tumor group, may explain the lack of significant difference found between the two groups (S2 Table).

**Table 3:** Correlation between colloid calcifications number and mRNA expression of different transporters, receptors or macromolecular inhibitors in 10 thyroid samples (Spearman test).

	Colloid calcification	
	Rho	P value
TRPV5	-0.721	**0.02**
TRPM6	-0.382	0.28
CaSR1	-0.455	0.19
VDR	-0.321	0.37
Cav1.3	-0.539	0.11
PMCA1	-0.200	0.58
MGLA	0.273	0.45
OPN	0.079	0.84
Calgranuline	0.600	0.07

Association of colloid calcifications number (dependent variable) with mRNA expression of candidate genes (normalized to 18S rRNA and thus expressed in arbitrary units) were tested using a spearman test.

However, as shown in Table 3, TRPV5 mRNA was strongly negatively correlated to colloid calcification number (Rho = -0.72, p = 0.02). Interestingly, TRPV5 mRNA was also positively associated with Cav1.3 and CaSR transcripts (p = 0.002 and p = 0.01 respectively). TRPV5 protein expression was confirmed by immunochemistry (Fig 4), with a strong cytoplasmic staining and in some thyrocytes an apical membrane staining (i.e. facing the colloid). Of notice, as shown in Fig 4G, a strong nuclear CaSR staining was detected on thyrocytes, whereas this receptor is localized in cell membranes in the kidney tubules (in the ascending limb of the loop of Henle) (Fig 4H).

#### Calcification and cancer

In our small series, there was no statistical correlation between different type of calcifications confirmed by FTIR and the tumor status. All samples containing psammoma bodies or capsule calcification, i.e. all calcium phosphates crystals, confirmed by μFTIR were detected in the tumor group samples, thus giving a 100% specificity. However,10 samples without calcium phosphate crystals were also classified in the tumor group, thus giving a poor sensitivity of 44% for this parameter (S1 Table).

Of notice, there was no significant difference for colloid calcification between the group Tumor and Non Tumor, neither for crystal composition and number, nor the thyrocytes’ phenotype (including Ki67, RUNX2, OPN, CD44, TRPV5 or CaSR). Besides, no link was found between colloid calcification number and patient’s age.

## Discussion

In our study, psammoma bodies and capsule calcifications were rare (5% and 9% of our sampling respectively), identified after careful proofreading, associated with papillary carcinoma and composed of calcium phosphate in 100% of cases. Conversely, colloid calcifications were initially underestimated, and were detected under a polarized microscope in 91% of cases and composed of calcium oxalate in 100% of cases with no phosphate compounds.

Psammoma bodies were reported to be associated with a malignant pathology [13,20,21] and a poor prognosis [22]. According to the current view, psammoma bodies would be ghosts of dead papillae [20,22,23]. However, in our series, the presence of psammoma bodies between the follicles or in the interlobular septa, which are usually described as the location of vessels or lymphatics [20] does not support this view. Three species of calcium phosphate: carbonated calcium apatite (CA), amorphous carbonated calcium phosphate (ACCP) and in one case small amount of whitlockite were detected, with a concentric and lamellar structure, suggesting growth lines and a slow process for crystal deposition. This new intriguing finding raises the issue of the mechanism of calcium phosphate deposits which remains unravel.

As 20 out of 56 samples without calcium phosphate crystals were also classified in the tumor group, malignity cannot be ruled out according to calcium phosphate content within crystals. Moreover, a previous study by Mathonnet et al [7] found calcium phosphate in nearly all their samples with only six cancers out of 34 samples. As samples in this latter study were selected upon the presence of macroscopic calcifications, we assume that most selected tissues contained capsule calcifications and not psammoma bodies.

Colloid calcifications are frequent and were found in 79% of autopsies [6]. They are described only in humans and not in animals [6,14] and were reported to contain calcium oxalate [5,6,9,24]. Using μFTIR spectrometry, we could further analyze their composition and found pure COM in 58% of cases and COD or COM + COD in all other cases. Calcium oxalate supersaturation process within colloid gel leading to a high prevalence of COM remains an open question. Indeed, oxalate concentration was reported as a major determinant for COM formation in urine [25]. Oxalate is usually found in mitochondria or in endoplasmic reticulum and its extracellular presence is intriguing. Iodine transporters (or other halogen elements such as bromine) in thyrocytes ensuring vectorial transport through colloid gel may potentially also transport other anions such as oxalate. Alternatively, some specific oxalate membrane transporters in the thyrocytes may be at play. Intriguingly, COD crystals had very different features from what is described in kidney stones: polarization properties conversely to COD in kidneys and unusual overlap COD sticks (Fig 3O) on FE-SEM analysis. EDX analysis confirmed calcium content in several samples thus ruling out binding of oxalate anions by other hypothetical cations. We speculate that the surrounding proteins (namely colloid proteins) may explain the discrepancy between thyroid and kidney COD structure. Calcium supersaturation within colloid gel accounting for COD crystals suggests also an unexpected efficient calcium transport into colloid follicles. Our results show the presence of TRPV5 (transient receptor potential vanilloid 5) in thyrocytes, a previously reported apical transcellular Ca(2+) transporter, highly selective for calcium, involved in calcium reabsorption in the kidney and in the intestine [26]. TRPV5 was described in many other tissues: mammary glands [27], parathyroid [28], placenta, prostate, salivary and sweat glands [29], but to our knowledge not yet in thyroid gland. As shown by RT-PCR, the number of colloid calcification was inversely correlated to the expression of the calcium transport TRPV5, thus suggesting that TRPV5 would prevent crystal deposits. However, the process of calcium transport into colloid follicle is beyond the scope of the present study and deserves a specific address in order to test among other issues TRPV5 regulation by CaSR and the vectorial calcium transport by TRPV5, Cav1.3 and PCAM1 among other candidates.

In our series, no correlation was found between colloid calcification number and age or gender, conversely to other studies [9,13,14,24]. In accordance with literature, no significant association between colloid calcifications and pathology was shown. Moreover, proliferation index was not associated with colloid calcification number; however, KI67 staining detects only ongoing cell division whereas crystals may be aggregated since months or years.

Limitation of the study: our observational cross sectional study does not allow the understanding of crystal deposits chronology. Whereas, prevalence, localization and characterizations of crystals within thyroids can be assessed, the relevance of thyrocyte proliferation (thyrocytes are usually known to have only few divisions in a lifetime) and differentiation can only be questioned. We speculate that upregulation of RUNX2, a specific osteoblastic transcription factor altogether with osteopontin (a macromolecular inhibitor) and its receptor CD44 could be key players for crystal prevention/aggregation. Some correlations could not be addressed due to small samples, such as correlations between expression of TPV5 mRNA expression or mRNA expression of various transporters, receptors or macromolecular inhibitors between the Tumor group and the non Tumor Group. Further studies deserve to be performed on a larger sample to address this issue.

Our study is the first to indirectly demonstrate a high concentration of calcium and oxalate within colloid gel altogether with the expression of several calcium transporters and potential crystal clearance (i.e; osteopontin and CD44), and as such should be considered preliminary to further studies focusing on follicle physiology for calcium and oxalate.

Our study shows that psammoma bodies are composed of concentric and lamellar structure of different species of calcium phosphate suggesting a slow process for crystal deposition outside colloid follicles. Conversely, colloid calcifications are very frequent and routinely underestimated unless a polarized microscopy examination is performed and composed of calcium oxalate in 100% of cases with no phosphate compounds. The presence of COM and COD crystals within colloid gel raises intriguing issues upon follicle physiology for calcium and oxalate vectorial transport. These findings pave the way for interesting perspective and suggest that ex vivo colloid follicles could be an interesting tool for functional studies.

## Supporting information

S1 Table Analysis of the presence of calcification (proved by μFTIR) among different histological structures according to the different pathologies. (DOCX)

S2 Table mRNA expression of various transporters, receptors and macromolecular inhibitors in 10 patients. Pathology*: TG = Tumor Group, NTG = Non Tumor Group.
(DOCX)

S3 Table Immunochemistry staining between Tumor Group and Non Tumor Group for OPN, CD44, Ki67, RUNX2, TRPV5 and CaSR. (DOCX)

S1 Fig The presence of calcium was further confirmed using electron diffraction X-ray microscope in all samples (EDX). a.EDX spectrum in a sample.
b.Mean calcium content in 13 samples. Presence of calcium was confirmed in all samples but its presence was heterogeneous in the same sample.
(TIF)
