# Supplementary material for: Localization and characterization of thyroid microcalcifications: A histopathological study
Source: PLoS One. 2019 Oct 24;14(10):e0224138. doi: 10.1371/journal.pone.0224138 (PMC6812851; doi:10.1371/journal.pone.0224138)
Supplement: S1 Table — (DOCX) [file pone.0224138.s001.docx]

|  | Psammoma bodies | | Capsule Calcification | | Colloid calcification | |
| --- | --- | --- | --- | --- | --- | --- |
|  | + | - | + | - | <=10 | >10 |
| Tumor Group (n=18) | 4 | 14 | 4 | 14 | 5 | 8 |
| Non Tumor Group (n=9) | 0 | 9 | 0 | 9 | 4 | 5 |

**S1 Table.** Analysis of the presence of calcification (proved by µFTIR) among different histological structures according to the different pathologies.
