# Supplementary material for: Localization and characterization of thyroid microcalcifications: A histopathological study
Source: PLoS One. 2019 Oct 24;14(10):e0224138. doi: 10.1371/journal.pone.0224138 (PMC6812851; doi:10.1371/journal.pone.0224138)
Supplement: S2 Table — Pathology*: TG = Tumor Group, NTG = Non Tumor Group. (DOCX) [file pone.0224138.s002.docx]

| Patient | Pathology* | Number of colloid calcifications | **mRNA expression** | | | | | | | | |
| --- | --- | --- | --- | --- | --- | --- | --- | --- | --- | --- | --- |
|  |  |  | **TRPV5** | **TRPM6** | **CaSR1** | **MGLA** | **OPN** | **Calgranuline** | **VDR** | **Cav1.3** | **PMCA1** |
| N°1 | TG | 55 | 0,004 | 0,0158 | 0,249 | 262,8 | 0,4407 | 1,204 | 0,1871 | 0,59 | 2,384 |
| N°2 | TG | 32 | 0,006 | 0,0244 | 0,213 | 216,1 | 0,2234 | 0,491 | 0,0786 | 0,44 | 1,823 |
| N°3 | TG | 17 | 0,007 | 0,0199 | 0,361 | 156,8 | 0,1282 | 0,135 | 0,1207 | 1,25 | 1,566 |
| N°4 | NTG | 56 | 0,005 | 0,0413 | 0,457 | 209 | 0,8841 | 2,501 | 0,4027 | 0,93 | 1,781 |
| N°5 | NTG | 25 | 0,009 | 0,0871 | 0,859 | 79,17 | 1,795 | 0,261 | 0,2048 | 1,39 | 2,874 |
| N°6 | NTG | 66 | 0,002 | 0,0190 | 0,0999 | 277,7 | 0,0434 | 2,959 | 0,0706 | 0,31 | 1,776 |
| N°7 | NTG | 10 | 0,018 | 0,0545 | 0,857 | 81,81 | 0,5502 | 0,271 | 0,4183 | 4,56 | 2,971 |
| N°8 | NTG | 3 | 0,006 | 0,0206 | 0,429 | 326,3 | 0,2744 | 0,612 | 0,1431 | 0,82 | 1,758 |
| N°ç | NTG | 40 | 0,007 | 0,0286 | 0,448 | 117,9 | 1,331 | 0,236 | 0,3387 | 1,6 | 2,765 |
| N°10 | NTG | 5 | 0,007 | 0,0781 | 0,638 | 86,53 | 0,0952 | 0,17 | 0,4241 | 2,1 | 3,282 |

**S2 Table.** S2 Table. mRNA expression of various transporters, receptors and macromolecular inhibitors in 10 patients. Pathology*: TG= Tumor Group, NTG=Non Tumor Group.
