# Supplementary material for: Localization and characterization of thyroid microcalcifications: A histopathological study
Source: PLoS One. 2019 Oct 24;14(10):e0224138. doi: 10.1371/journal.pone.0224138 (PMC6812851; doi:10.1371/journal.pone.0224138)
Supplement: S3 Table — (DOCX) [file pone.0224138.s003.docx]

|  |  | Tumor Group | Non Tumor Group | p |
| --- | --- | --- | --- | --- |
| OPN | n=57 | 24% | 28% | p=0.60 |
| CD44 | n=57 | 47% | 32% | p=0.052 |
| Ki67 | n=30 | 38% | 32% | p=0.54 |
| RUNX2 | n=36 | 39% | 46% | p=0.49 |
| TRPV5 | n=45 | 26% | 14% | p=0.22 |
| CaSR | N=12 | 65% | 100% | p=0,09 |

**S3 Table**. Immunochemistry staining between Tumor Group and Non Tumor Group for OPN, CD44, Ki67, RUNX2, TRPV5 and CaSR.
