# Supplementary figures and images for: Localization and characterization of thyroid microcalcifications: A histopathological study
Source: PLoS One. 2019 Oct 24;14(10):e0224138. doi: 10.1371/journal.pone.0224138 (PMC6812851; doi:10.1371/journal.pone.0224138)

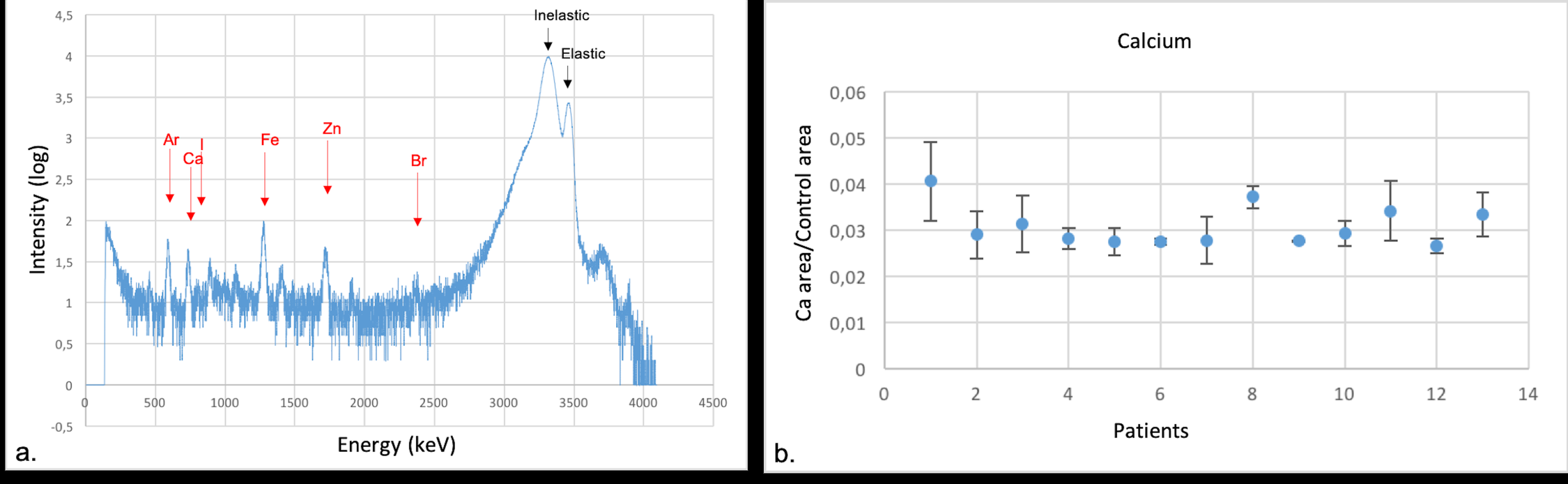

Supplement: S1 Fig — a.EDX spectrum in a sample. b.Mean calcium content in 13 samples. Presence of calcium was confirmed in all samples but its presence was heterogeneous in the same sample. (TIF) [file pone.0224138.s004.tif]
